# Correlation of outcome measures with epidemiological factors in thoracolumbar spinal trauma

**DOI:** 10.4103/0019-5413.36989

**Published:** 2007

**Authors:** Bidre Upendra, Bijjawara Mahesh, Lalit Sharma, Pankaj Khandwal, Abrar Ahmed, Buddhadev Chowdhury, Arvind Jayaswal

**Affiliations:** Department of Orthopaedics, All India Institute of Medical Sciences, New Delhi, India

**Keywords:** Epidemiology, thoracolumbar fractures, trauma-care system

## Abstract

**Background:**

The epidemiological data of a given population on spinal trauma in India is lacking. The present study was undertaken to evaluate the profile of patients with thoracolumbar fractures in a tertiary care hospital in an urban setup.

**Materials and Methods:**

Four hundred forty patients with thoracolumbar spinal injuries admitted from January 1990 to May 2000 to the All India Institute of Medical Sciences were included in the analysis. Both retrospective data retrieval and prospective data evaluation of patients were done from January 1998 to May 2000. Epidemiological factors like age, sex and type of injury, mode of transport, time of reporting and number of transfers before admission were recorded. Frankel's grading was used to assess neurological status. Functional assessment of all patients was done using the FIM™ instrument (Functional Independence Measure). Average followup was 33 months (24-41 months).

**Results:**

Of the 440 patients, females comprised 17.95% (n=79), while 82.04% (n=361) were males. As many as 40.9% (n=180) of them were in the third decade. Fall from height remained the most common cause (n=230, 52.3%). Two hundred sixty (59.1%) patients reported within 48 hours. Thirty-two (7.27%) patients had single transfer, and all 32 showed complete independence for mobility at final followup. 100 of 260 (38.5%) patients reporting within 48 hours developed pressure sores, while 114 of 142 (80.28%) patients reporting after 5 days developed pressure sores.

**Conclusion:**

The present study highlights the magnitude of the problems of our trauma-care and transport system and the difference an effective system can make in the care of spinal injury patients. There is an urgent need for epidemiological data on a larger scale to emphasize the need for a better trauma-care system and pave way for adaptation of well-established trauma-care systems from developed countries.

Traumatic paraplegia in the young often results in profound change in the life of the injured and also his or her family. These injuries also have tremendous social costs due to prolonged health-care treatment, rehabilitation and productivity loss.^1^,^2^ The annual incidence of traumatic spinal cord injury (SCI) in developed countries varies from 11.5 to 53.4 per million of population.^3^ Deaths after admission for acute SCI range from 4.4% to 16.7%.^4^ There is a dearth of such data on the national scale in developing countries like India.

Epidemiology helps us understand the extent of the problem of traumatic paraplegia and the importance of primary care. Epidemiologic studies provide estimates of incidence and prevalence, identify high-risk groups and thus provide insight into priorities for resource allocation, etiologic research and prevention efforts. They also provide a baseline from which to gauge the effectiveness of trauma-care systems involved in transport of the victims to their definitive care in specialized centers. Further, evaluation of the functional outcome of these patients in relation to the modes and timing of transport can provide invaluable insight into the means to improve the existing trauma-care facilities. However, unlike western literature, the epidemiological data of a given population in India is lacking. The present study was undertaken to evaluate the profile of patients with thoracolumbar fractures coming to a tertiary care hospital in an urban setup. This study is not a true epidemiological study as it does not represent the incidence of thoracolumbar injuries in the general population in a geographic area. The study aims to correlate the outcome of spinal cord injury with various epidemiological factors like type of injury, mode of transport, time of reporting, etc.

## MATERIALS AND METHODS

Four hundred forty patients with unstable thoracolumbar spinal injuries admitted from January 1990 to May 2000 to the All India Institute of Medical Sciences constituted the clinical material. Both retrospective and prospective data of the patients from January 1998 to May 2000 were retrived, documented and evaluated. Epidemiological factors like age, sex and type of injury, mode of transport, time of reporting and number of transfers before admission were recorded. Frankel's grading^5^ was used to assess neurological status. Effect of the above factors on neurological recovery and incidence of complications at two years were evaluated.

Functional assessment of all patients was done using the FIM™ instrument^6^ (Functional Independence Measure), which is a standard tool used to measure function in people undergoing rehabilitation. Patient performances on the 18 activities are rated on a 7-level scale, with ‘1’ indicating total assistance and ‘7’ indicating complete independence (timely, safely). Functional independence staging (FIS), introduced by Stineman *et al.,*^7^ addresses the limitations experienced in applying continuous, aggregate FIM scores in clinical practice. He described a system that used each of the four domains of function to define profiles or stages of function. The domains include activities of daily living (ADLs) (FIM items for eating, grooming, bathing, dressing upper body, dressing lower body, toileting), sphincter management (FIM items for bladder management, bowel management), mobility (FIM items for bed, chair and wheelchair transfer; toilet transfer; tub or shower transfer; walking or wheelchair mobility) and executive function (FIM items for comprehension, expression, social interaction, problem-solving, memory). Only the mobility domain was evaluated in this study at the time of admission and the last followup from the records available. Each domain is comprised of seven stages. The stages suggest the average amount of assistance needed by the patient and the amount of effort required by the patient within each domain. For example, stage 1 in any domain equates with the patient being able to provide less than 25% of the effort required to accomplish the tasks in the domain; stage 4 indicates a minimal amount of assistance from another person and an effort of 75% on the part of the patient and stage 7 indicates full independence.

Two hundred eighty-eight patients with unstable injuries (unstable burst, fracture dislocation) or progressive neurological deficit (correlating with >50% canal compromise on axial CT scans) were managed with surgical decompression and stabilization. Anterior decompression and instrumentation was done when posterior ligamentous complex (PLC) was intact on MRI, and postero-lateral decompression of the cord with posterior instrumentation with interbody support using interbody cages was done with three column injuries with PLC disruption. The rest of the patients were managed conservatively with 4 weeks of bed rest with water/ ripple mattress and log-rolling. Mobilization on wheel chair with thoracolumbar jacket was started after four weeks. Patients were followed up at 3 months, 6 months, 1 year and 2 years for level of rehabilitation and neurological function, along with complications. Average followup was 33 months (24-41 months).

## RESULTS

Of the 440 patients, females comprised 17.95% (n=79), while 82.04% (n=361) were males (M:F – 4.58:1). 180 (40.9%) of them were in the third decade, 84 (19.09%) of the patients were aged between 11 and 20 years of age, 44 (10%) of the patients belonged to the fourth decade, 72 (16.36%) were between 41 and 50 years, 48 (10.9%) were in the sixth decade and 12 (2.72%) were above the age of 60 years.

Fall from height remained the most common cause, with 230 (52.3%) patients, followed by road traffic accident (RTA) (n=169; 38.4%), various forms of violence (n=22; 5.0%) and gun-shot injuries (n=19; 4.3%).

Two hundred sixty (59.1%) of the patients reported within 48 hours, 38 (8.6%) reported between two and four days, 52 (11.8%) between five and 10 days and 90 (20.4%) came 10 days after the injury. Two hundred ten (47.7%) patients were transported by ambulance, while the rest were brought by other means (in rickshaws, cars, jeeps, etc.).

Thirty-two (7.27%) patients had single transfer (32 transfers), 320 (72.7%) patients were transferred 2-4 times (mean 3.1 times; 992 transfers) and 88 (20%) patients were transferred 5-7 times (mean 5.33 times; 469 transfers) before reaching the definitive care center; the mean transfer rate was 3.4 per person.

Frankel grading showed that on admission 294 patients (66.8%) were in Grade-A; 82 patients (18.63%), Grade-B; 28 (6.36%), Grade-C; 16 (3.6%), Grade-D; and 20 (4.54%) were in Grade-E with no deficit. Sixteen (3.63%) patients succumbed to other injuries or complications in few weeks/months of injury. At the final followup, the neurological status was Grade-A in 204 patients, Grade-B in 20, Grade-C in 63, Grade-D in 55 and Grade-E in 82 patients out of remaining 424 patients. Seventy-four patients out of 294 (25.1%) with Frankel-A showed some neurological improvement and 16 patients died; 11 to Frankel-B (3.74%), 36 to Frankel-C (12.24%), 20 to Frankel-D (6.8%) and seven to Frankel-E (2.38%) [Table 1].

**Table 1:** Frankel grading at admission and final followup

Frankel Grade	Admission	Final followup (16 expired)	Difference
Grade-A	294	204	-90 (74 improved, 16 died)
Grade-B	82	20	-62
Grade-C	28	63	+35
Grade-D	16	55	+39
Grade-E	20	82	+62
Total	440	424	-16

Patients with single transfer, ambulance transfers and those reporting within 48 hours showed better neurological recovery [Table 2], but on statistical analysis there was no significant correlation found between neurological recovery and the time of admission, mode and number of transports, surgical or conservative management.

**Table 2:** Frankel grade at admission in relation to the number of transports

Frankel Grade	Single transfer n=32 No. (%)	2-4 transfers n=320 No. (%)	5-7 transfers n=88 No. (%)
Grade-A n=294	0	220 (68.75)	74 (84.09)
Grade-B n=82	11 (34.37)	67 (20.93)	4 (4.54)
Grade-C n=28	10 (31.25)	13 (4.06)	5 (5.68)
Grade-D n=16	8 (25.0)	4 (1.25)	4 (4.54)
Grade-E n=20	3 (9.37)	16 (5.0)	1 (1.14)
Total n=440	32	320	88

The mobility functional independence staging (FIS) at final followup showed correlation with the mode of transport, time of transport and number of transports. All 32 (100%) patients with single transfer showed complete independence for mobility at final followup, while 190 (59.4%) of 320 patients who were transported between two and four times recovered to either complete or modified independence (*P* < 0.01). As many as 39 (53.4%) of 260 of patients who reported within 48 hours recovered to either complete or modified independence, while 22 (24.5%) of 90 showed recovery to complete or modified independence on FIS in those who reported after 10 days [Table 3]. Ambulance transfer showed 140 (66.7%) out of 210 patients recovered to complete or modified independence, while 90 (39.2%) of 230 those transported by other means had modified independence [Table 4]. Out of 288, 166 (57.6%) patients recovered to complete or modified independence after surgical management [Table 4], whereas conservative management resulted in 71 (46.7%) of 152 patients attaining complete or modified independence (*P* > 0.1).

**Table 3:** Functional independence staging in relation to no. of transports and reporting time

	Single transfer no=32 No. (%)	2-4 transfers no=320 No. (%)	5-7 transfers no=88 No. (%)	Rep.time <48 hrs no= 260 No. (%)	Rep.time 2-4 days no=38 No. (%)	Rep.time 5-10 days no= 52 No. (%)	Rep.time >10 days no=90 No. (%)
Complete independence/modified independence (level-7,6)	32 (100)	190 (59.4)	24 (27.27)	139 (53.4)	18 (47.36)	19 (36.5)	22 (24.44)
Modified dependence (level-5,4,3)	0	114 (35.62)	52 (59.09)	106 (40.7)	11 (28.9)	18 (34.6)	54 (60.0)
Complete dependence (levels-2,1)	0	16 (5)	12 (13.63)	15 (5.77)	9 (23.68)	15 (28.84)	14 (15.5)

**Table 4:** Functional independence staging in relation to mode of transport and surgical/conservative management

	Transport-ambulance no= 210 No. (%)	Transport-other means no= 230 No. (%)	Surgical group (no=288) No. (%)	Conservative (no=152) No. (%)
Complete independence/modified independence (level-7,6)	140 (66.7)	90 (39.1)	166 (57.6)	71 (46.7)
Modified dependence (level-5,4,3)	52 (24.76)	112 (48.69)	101 (35.07)	52 (34.21)
Complete dependence (levels-2,1)	18 (8.57)	28 (12.17)	21 (7.29)	29 (19.08)

In 100 of 260 (38.5%) patients reporting within 48 hours developed pressure sores, while 114 of 142 patients (80.28%) of patients reporting after five days developed pressure sores (*P* < 0.01). Ninety (31.2%) patients undergoing surgery developed pressure sores; of which 44 (48.8%) had reported after five days of injury, as against 102 (67.1%) in the conservative group. Sixty-nine patients (32.9%) transported via ambulance had pressure sores, while 122 (53.04%) out of 230 patients transported by other means developed pressure sores (*P* > 0.05).

## DISCUSSION

Thoracolumbar fractures have a significantly distressing impact at the individual level; and are also detrimental to the society, with significant financial drain and loss of human resources. This is compounded by the fact that the majority (73%) of thoracic spine fractures produce complete spinal cord injury.^8^ The annual incidence of SCI in developed countries varies from 11.5 to 53.4 per million population.^3^ Such data, however, are not yet available in our country. The present study does not give the true epidemiological data, like the incidence and prevalence in the population as a whole, but gives valuable information about the injury pattern and the trauma-care and transport systems in an urban setup.

The present study shows that thoracolumbar spinal injuries are most common between 21 and 30 years of age. This correlates well with similar findings in most epidemiological studies.^4^,^9^,^10^ The sex distribution in this study shows a trend towards increasing number of spinal trauma in females. Indian epidemiological studies^11^,^12^ in the rural setup have shown a high preponderance of spinal trauma involving males, with the ratio of male to female ranging from 9:1 to 13.5:1. The ratio of male to female of 4.5:1 in this study compares well with western literature.^13^

The most common mode of injury was fall from height. This correlates with the previous Indian studies by Chacko *et al.*^11^ and Shanmugasundaram,^12^ but most of the western studies have RTA as the leading cause of spinal trauma.^14^ However, there is a trend towards increasing incidence of RTA as compared to previous Indian studies.^11^,^12^ The increased incidence of violence and gun-shot injuries reflects the increasing unrest in the urban life styles with increase in crime rate. The incidence of spinal cord injuries due to violence in the United States is about 11% but is only 4% in Canada, corresponding to the extent of social violence.^15^

There was no significant correlation found between neurological recovery and the time of admission, mode and number of transports, surgical or conservative management; and the initial neurological status largely determined the amount of further recovery, as has been reported widely in the literature.^16^

The most striking difference between the present study and the western literature is seen in the mode of transport of the spinal trauma victim to the definitive care center and the time of patient-reporting. Most developed countries possess an effective trauma-care and transport system, which efficiently transports the victims, usually within the first 12–24 hours, to the definitive care centers via well-equipped ambulance.^17^ The lack of any such effective transport system, even in the urban setup of this study, is very glaring. Only 59.1% of the victims reached the institute within the first 48 hours, and only 47.7% of the patients with spinal trauma were transported in an ambulance. It is also evident from this study that most of the victims were transported 2–4 times between various health-care centers before reaching the definitive spinal-care center. Only 7.27% of the patients were transferred directly to the institute, and the average number of transfers was 3.4 per patient. This is an embarrassing situation in the trauma-care system that we find ourselves in, even in the urban setup. The mobility functional independence staging (FIS) at final followup showed correlation with the mode of transport, time of transport and number of transports. However, the neurological status at the time of initial presentation is important before concluding that decreased number of transports resulted in better functional status. On analysis the data showed that 100% [32 of 32] of patients reporting to the institute with single transfer had partial or no neurological deficit as against 100 of 320 [31.24%] patients with 2–4 transfers and 15.91% [14 of 88] with 5-7 transfers. This signifies that the excellent final functional status achieved in single-transfer patients was directly related to the partial spinal cord injury at the time of admission. However, this also brings out the fact that as the number of transfers increased, the percentage of patients reporting with complete paraplegia [Frankel-A] significantly increased [Table 2]. This has great impact on the final functional status achieved by the patient, as the neurological recovery to ambulatory status in patients with Frankel-A is around 3–8% and that in patients with partial cord injury [Frankel-B, C, D] is around 30–40%. Hence with lesser number of transfers, there would be lesser chances of secondary injury^18^ to spinal cord, leading to better neurological status at presentation, which then translates into better functional recovery. The importance of an early and effective ambulance transfer of the spinal trauma patient cannot be overemphasized. It is now recognized that one of the major factors for good outcome in spinal injury patients is the prehospital care and timely transport to the definitive care center.

Acute spinal cord injury is a two-step process involving primary and secondary mechanisms.^18^ The primary mechanism involves the initial mechanical injury due to local deformation and energy transformation, whereas the secondary mechanism encompasses a cascade of biochemical and cellular processes that are initiated by the primary process and may cause ongoing cellular damage and even cell death.^19^ The important causes for secondary spinal cord injury are hypoxia, hypo/hyperthermia, hypotension and injudicious movement of the unstable spine leading to worsening compression. Therefore, prehospital care with proper spinal stabilization, prevention of hypotension and oxygen therapy would contribute significantly in minimizing secondary injury to the spinal cord. The results of this study reinforce the importance of early and proper ambulance transfers in improving the functional outcome in thoracolumbar fracture patients. Patients with single transfers and ambulance transfers attained a better functional status than others [Tables 3 and 4]. Studies by Chacko *et al.* also show a poor ambulance transfer rate of 34% in the rural setup. Most of the victims are transferred by relatives of the patient in cars, rickshaws, jeeps, etc., with little knowledge of the importance of spinal immobilization. In comparison, most developed countries have >90% ambulance transfers of the victim to the definitive care center within 12-24 hours, with trained paramedical personnel.^14^ The reasons stated for the lack of such effective functioning of trauma-care systems in our country are the huge population to be catered to; and lack of education, awareness, enough trained paramedical personnel and infrastructure. India, now, is recognized worldwide as a fast-growing economy and huge infrastructure undertakings are already a reality in urban transport systems. In this context, the dream of an effective trauma-care system needs to be given a genuine thought and opportunity considering the far-reaching effects it can have on the outcome of trauma victims at the individual level and at the social level as a whole in terms of loss of human resources and economic burden that such injuries cause. The present study highlights the magnitude of the problems of our trauma-care and transport system in particular and the difference an effective system can make in the care of spinal injury patients. Therefore, there is an urgent need for epidemiological data on a larger scale to emphasize the need for a better trauma-care system which can morally compel, and pave the way for, adaptation of well-established trauma-care and transport systems such as those already functioning in many developed countries.
